# Impaired Subjective Visual Vertical and Increased Visual Dependence in Older Adults With Falls

**DOI:** 10.3389/fnagi.2021.667608

**Published:** 2021-06-11

**Authors:** Milda Totilienė, Virgilijus Uloza, Vita Lesauskaitė, Gytė Damulevičienė, Rima Kregždytė, Diego Kaski, Ingrida Ulozienė

**Affiliations:** ^1^Department of Otorhinolaryngology, Lithuanian University of Health Sciences, Kaunas, Lithuania; ^2^Department of Geriatrics, Lithuanian University of Health Sciences, Kaunas, Lithuania; ^3^Department of Preventive Medicine, Lithuanian University of Health Sciences, Kaunas, Lithuania; ^4^Department of Clinical and Movement Neurosciences, University College London, London, United Kingdom

**Keywords:** subjective visual vertical, older people, falls, virtual reality, visual dependence

## Abstract

Aging affects the vestibular system and may disturb the perception of verticality and lead to increased visual dependence (VD). Studies have identified that abnormal upright perception influences the risk of falling. The aim of our study was to evaluate subjective visual vertical (SVV) and VD using a mobile virtual reality-based system for SVV assessment (VIRVEST) in older adults with falls and evaluate its relationship with clinical balance assessment tools, dizziness, mental state, and depression level. This study included 37 adults >65 years who experienced falls and 40 non-faller age-matched controls. Three tests were performed using the VIRVEST system: a static SVV, dynamic SVV with clockwise and counter-clockwise background stimulus motion. VD was calculated as the mean of absolute values of the rod tilt from each trial of dynamic SVV minus the mean static SVV rod tilt. Older adults who experienced falls manifested significantly larger biases in static SVV (*p* = 0.012), dynamic SVV (*p* < 0.001), and VD (*p* = 0.014) than controls. The increase in static SVV (odds ratio = 1.365, *p* = 0.023), dynamic SVV (odds ratio = 1.623, *p* < 0.001) and VD (odds ratio = 1.460, *p* = 0.010) tilt by one degree significantly related to falls risk in the faller group. Fallers who had a high risk of falling according to the Tinetti test exhibited significantly higher tilts of dynamic SVV than those who had a low or medium risk (*p* = 0.037). In the faller group, the increase of the dynamic SVV tilt by one degree was significantly related to falls risk according to the Tinetti test (odds ratio = 1.356, *p* = 0.049). SVV errors, particularly with the dynamic SVV test (i.e., greater VD) were associated with an increased risk of falling in the faller group. The VIRVEST system may be applicable in clinical settings for SVV testing and predicting falls in older adults.

## Introduction

The perception of verticality relates to a subject’s ability to determine an Earth vertical line without external reference cues and is a critical component of normal balance and gait (Barr et al., 2016; Dieterich and Brandt, 2019). The most frequent test used to assess perceived verticality is the subjective visual vertical (SVV) test (Mueller et al., 2020), and has been used as a surrogate measure of peripheral or central vestibular impairments (Baier et al., 2016). Aside from a range of distinct vestibular pathologies, aging has been shown to alter vestibular function, including SVV perception (Jahn, 2019). Age-related vestibular changes include hair cell loss, neuronal loss, degeneration of the vestibular ganglion and nerve, and reduced blood flow to the inner ear (Zalewski, 2015). Deterioration of the function of the otolith organs includes an age-dependent reduction in afferent signals to the integrating centers for SVV within the central nervous system and consequently reduced sensitivity to gravity and linear acceleration (Walther and Westhofen, 2007). This leads to an increase in weighting from sensory systems that provide more reliable information, such as vision (Lee, 2017a, b). The increased reliance on visual stimuli is termed visual dependence (VD).

Understanding the role of vestibular dysfunction across age has important implications for maintaining upright balance, and prevention of falls among older individuals (Tjernström et al., 2016). Indeed, according to the WHO global report, 28–35% of people aged >65 years fall each year, and this prevalence increases to 32–42% for people >70 years old, with associated health, social and economic burden (Kannus et al., 2005; WHO, 2008). The major factors responsible for the increase in fall risks in older adults are balance and gait control and musculoskeletal, cardiovascular, vestibular, somatosensory, and visual dysfunction (Cho et al., 2004; Ekvall Hansson and Magnusson, 2013). Dizziness itself in older age is a strong predictor of falls (Agrawal et al., 2009). Moreover, aging effects upon cognition and associated psychological factors, such as depression and anxiety, may also contribute to falls (Laurence and Michel, 2017).

The present comparative cross-sectional study aimed to assess SVV and VD using an established mobile virtual reality-based system for subjective visual vertical assessment (VIRVEST) in older adults with falls and evaluate its relationship with the results of other balance assessment tools, dizziness symptom burden, mental state, and psychological factors.

## Materials and Methods

### Participants

This study enrolled 37 adults 65 years or older (faller group) who were hospitalized in the Department of Geriatrics of Clinical Hospital of Kaunas, Lithuania, between 2017 and 2019 and 40 non-faller age-matched controls (non-faller group) in the Department of Otorhinolaryngology, Lithuanian University of Health Sciences, Kaunas. We carried out thorough clinical histories in all patients and controls, including detailed information regarding impairments of vision, hearing, and inner ear diseases, which were not found in the patients or controls. Participants with significant ophthalmological, otological, neurological, or musculoskeletal impairments were excluded from this study. The faller group participants were not hospitalized as a consequence of falls but were selected from this department for assessment convenience and were medically stable. Older people considered at risk of falling and hospitalized in our Department of Geriatrics were routinely asked whether they have fallen in the past year and asked about the frequency, context, and characteristics of the fall. In accordance with NICE guidelines (NICE, 2015), a fall was defined as a sudden change in the person’s current position to a position lower than their height. At least one indoor or outdoor fall related to dizziness, weakness, or tripping, but unrelated to violence or use of any physical force, was counted as a fall (NICE, 2015). Precise data on patients who had one fall and those who have had more than one fall was not available as this was not one of the aims of this study. Therefore, we did not split these up into separate groups for this particular research. In line with NICE guidelines (NICE, 2013), we performed multifactorial fall risk assesment, including the Timed Up and Go test (TUG), the Four-Stage Balance test, the Five Times Sit To Stand (5TSTS), and Tinetti test in all patients. All control participants (non-fallers) underwent a detailed clinical history to ensure there had been no preceding falls over the last year. Control non-faller group individuals were volunteers, they had normal caloric (air irrigation at 44°C and 30°C, over 40 s and 3 min between irrigations) and cVEMP function (acoustic stimulus intensity of 100 dB and a frequency of 500 Hz) prior to the experimental testing. The mean ± SD of P1 latency (msec) for the left ear was 12.44 ± 1.4, for the right ear was 12.37 ± 1.24. For N1 mean latency (msec) for the left ear was 22.60 ± 1.26, 22.32 ± 1.35 for the right. The mean ± SD for amplitude ratio was 0.17 ± 0.9. cVEMP responses were considered abnormal when asymmetrical (ratio of 30% or greater), where P1 was absent, or P1 latency was outside of published normative values (McCaslin et al., 2013, 2014). oVEMPs were not carried out, although subjective visual vertical is an established test for utricular function, and therefore it was not deemed necessary to expose individuals to further vestibular testing. Video head impulse testing was not available in the study center and was not performed in the control group. We were not able to perform videonystagmography with caloric testing and cVEMP testing in the faller group because people were hospitalized in the Department of Geriatrics where instrumental assessment was not available. This was only available for the control group. MRI scans were not a routine part of the geriatric assessment for past falls. Fallers who were unable to walk without aids or assistance and adults who scored 10 or less on the Mini-Mental State Examination (MMSE) were excluded (to ensure task comprehension). Optical Frenzel glasses were used to look for spontaneous and gaze-evoked nystagmus (Ny). Clinical/balance tests were only performed in the faller group as the control group was deemed to consist of otherwise healthy individuals.

### Methods

**Figure 1:**
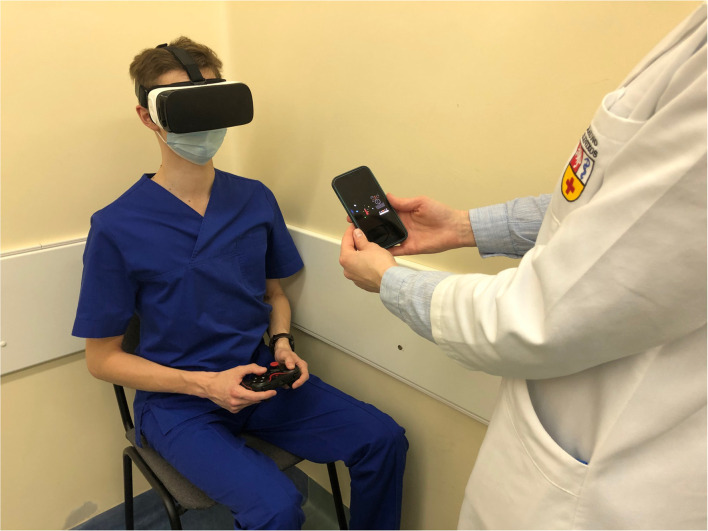
Testing with the VIRVEST system (consent statement obtained).

Subjective visual vertical test with the VIRVEST system includes a Samsung Gear VR headset, two smartphones (Samsung Galaxy S7), a software application, and a gamepad (Red Samurai gamepad, GameStop Corp. Inc., USA; Figure 1). One smartphone with a mobile application for virtual reality was used to observe the 3D stimulus, and another mobile device with a software application was used for the assessment of the participant who controlled and performed the test. The system enables us to observe a 3D stimulus presented across three separate conditions: a static SVV, a dynamic clockwise SVV, and a dynamic counter-clockwise SVV. The virtual reality environment is controlled by the participant using a Bluetooth-connected gamepad (Ulozienė et al., 2017).

The non-faller and faller groups completed the SVV tests using a VIRVEST system. The individuals’ task was to align the arrow to their perceived vertical in a black stationary background and in a moving background. For the dynamic test, 10 colored spheres were superimposed upon the black background, rotating in either clockwise or counter-clockwise directions. Based on pilot data, the background motion was set to a constant velocity of 10^0^/s given that higher values tended to interfere with accurate verticality perception in healthy controls. Six trials were completed for each participant, taking on average less than 1 min. Whilst having the potential to induce vection, dynamic SVV error rate does not differ across a short intertrial interval (<1 min) in controls, such that we did not observe learning or trial biases. The VIRVEST system has been previously validated in a healthy population and in patients with multiple sclerosis (Ulozienė et al., 2017, 2020). Three parameters were evaluated: static SVV, dynamic SVV, and VD. Visual dependence was calculated as the mean of the absolute values of the rod tilt from each trial of dynamic SVV minus the static SVV trials. Other studies also used a similar measure of VD with computerized (Cousins et al., 2014; Ulozienė et al., 2020) and noncomputerized versions (Roberts et al., 2016; Bednarczuk et al., 2020) of SVV.

We segregated patients into high, medium, and low risk of falling (continuous data for the TUG were converted to categorical data) as this has been purported to be more clinically meaningful. Thus, fallers who completed the Timed Up and Go (TUG) test in less than 12 s were considered to have a low risk of falling, 12–20 s was considered a medium risk of falling, and greater than 20 s was considered a high risk of falling (Podsiadlo and Richardson, 1991).

To assess the static balance, the Four-Stage Balance test was performed. Not being able to hold the tandem stance for 10 s was an indication of an increased risk for falling (Phelan et al., 2015). According to these results, fallers were divided into two groups: (1) fallers who had a low risk of falling and (2) fallers who had a high risk of falling.

Fallers who took longer than 10 s to perform the Five Times Sit To Stand (5TSTS) test or were not able to stand up five times, were considered to be at high risk for falling (Guralnik et al., 1994). According to the results of this test, fallers were divided into two groups: (1) fallers who had a low risk of falling and (2) fallers had a high risk of falling.

Balance abilities were evaluated in a chair, standing, and while walking during the Tinetti test. Fallers who collected scores of 18 or less were considered to have a high risk of falling, those with scores from 19 to 23 were considered to have a moderate risk of falling, and those with scores of 24 or more were considered to have a low risk of falling (Tinetti et al., 1986).

In the present study, we used the shortened form of the Geriatric Depression Scale (GDS), which is comprised of 15 items. Based on GDS scores, continuous data were converted to categorical data in order to make these continuous data of more direct clinical relevance and were divided as follows: (1) no depression ≤5; (2) probable depression, 5–10; and (3) depression >10 (Yesavage and Sheikh, 1986).

The MMSE was used to measure cognitive impairment in the faller group (Folstein et al., 1975). According to the MMSE results, fallers were divided into two groups: (1) fallers who had no cognitive impairment with scores of 25 or more; (2) fallers who had mild or moderate cognitive impairment with scores from 11 to 24.

Fallers evaluated their perceived dizziness and its impact on their daily life by completing the Dizziness Handicap Inventory (DHI) questionnaire (Mutlu and Serbetcioglu, 2013; Valančius et al., 2019).

As the VIRVEST system has not been previously validated in older people, we assessed its usability with the System Usability Scale (SUS) questionnaire. The SUS is a global, effective, and reliable tool for measuring the usability of a wide variety of products, including websites, applications, cell phones, and TV applications (Brooke, 1996; Bangor et al., 2008). Total scores correspond with seven qualitative adjective ratings, ranging from "worst imaginable” at the low end to “best imaginable” at the high end or range 0–64 (not acceptable), 65–84 (acceptable), and 85–100 (excellent) (Bangor et al., 2008; Mclellan et al., 2012).

### Statistical Analysis

Data analysis was performed using IBM SPSS Statistics 23.0 (IBM Corp., Armonk, NY). Quantitative data were tested for the distribution of normality. The Shapiro-Wilk test showed that the data distribution was not normal for all measurements. Nonparametric Mann-Whitney U or Kruskal-Wallis tests were applied to compare quantitative data among independent groups. Continuous variables (Static SVV, Dynamic SVV, VD) were compared across groups of the categorical variable consisting of more than two groups (TUG, DHI, GDS) applying the general Kruskal-Wallis test. In our study p-values of all simultaneous Kruskal-Wallis tests were more than 0.05, therefore *post hoc* tests (multiple pairwise comparisons) were not performed. Continuous variables (Static SVV, Dynamic SVV, VD) were compared between groups of the categorical variable consisting of two groups (“Faller/Non-faller”, “Tinetti group”) applying the Mann-Whitney U test. Logistic regression analysis was applied to evaluate factors involved in fall risk. Due to the small sample size, only one independent variable could be included in the model. Future studies may consider a multiple regression analysis across a larger population. Applying logistic regression we evaluated the odds of falling using the Tinetti test results and the history of falls. VD was calculated as the mean of absolute values of the rod tilt from each trial of dynamic SVV minus the mean static SVV rod tilt. Differences and relationships were considered statistically significant if *p* < 0.05.

## Results

The main demographic data are presented in Table 1 and clinical data of the faller group in Table 2. The faller and non-faller groups did not differ with respect to age (Mann-Whitney *U* = 588.0; *p* = 0.12) or gender (*χ*^2^ = 0.24; *p* = 0.62).

Results of the SVV tests in the faller and non-faller groups.

**Table 1:** Demographical data of the faller and non-faller group.

Characteristic	Faller group	Non-faller group
	*N* = 37	*N* = 40
Gender (M/F)	11/26 (30%/70%)	14/26 (35%65%)
Age, years (mean ± SD)	80.86 ± 6.84	78.70 ± 5.85

**Table 2:** Demographical and clinical data of the faller group.

Characteristic	Faller group
	*N* = 37
Gender (M/F)	11/26 (30%/70%)
Age, years (mean ± SD)	80.86 ± 6.84
Body mass index kg/m2 (mean ± SD; range)	26.81 ± 4.39; 19–36
DHI score:
mild handicap	6 (16%)
moderate handicap	10 (27%)
severe handicap	21 (57%)
TUG:
low risk of falling	4 (11%)
medium risk of falling	10 (27%)
high risk of falling	23 (62%)
Tandem stance:
low risk of falling	16 (43%)
high risk of falling	21 (57%)
5TSTS:
low risk of falling	2 (5%)
high risk of falling	35 (95%)
Tinetti test:
moderate or low risk of falling	13 (35%)
high risk of falling	24 (65%)
GDS:
no depression	13 (35%)
probable depression	18 (49%)
depression	6 (16%)
MMSE:
no cognitive impairment	10 (27%)
mild or moderate cognitive impairment	27 (73%)
Ny evaluation with optical Frenzel glasses:
spontaneous Ny	0 (0%)
gaze-evoked Ny	0 (0%)

*Abbreviations: DHI, Dizziness Handicap Inventory; TUG, Timed Up and Go; 5TSTS, Five Times Sit-to-Stand; GDS, Geriatric Depression Scale; MMSE, Mini-Mental State Examination; Ny, Nystagmus*.

**Figure 2:**
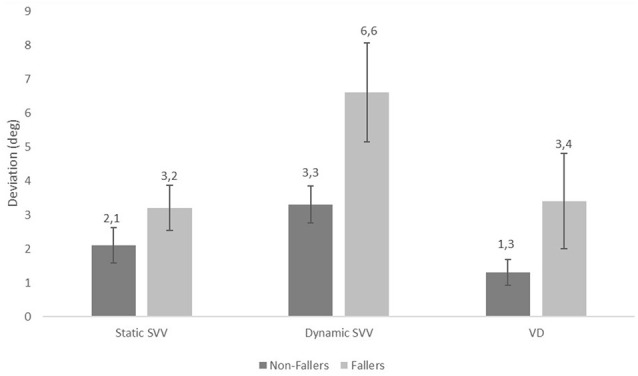
The mean values of static Subjective Visual Vertical (SVV), dynamic SVV tilts, and Visual Dependence (VD) assessment in the non-faller and faller groups. Vertical columns show the mean of the measurements, and vertical bars show the 95% confidence *intervals* (*p* = 0.012 for static SVV tilts, *p* < 0.001 for dynamic SVV tilts, *p* = 0.014 for VD).

The mean values of static SVV (*p* = 0.012), dynamic SVV (*p* < 0.001) tilts, and VD (*p* = 0.014) were significantly higher in the faller group than in the non-faller group (Figure 2). Moreover, we evaluated how the risk of falls was related to the results of static SVV, dynamic SVV tilts, and VD assessment compared to the history of falls. Univariate logistic regression analysis was applied for each quantitative predictor. We found that the increase in static SVV tilt (odds ratio = 1.365, *p* = 0.023), dynamic SVV (odds ratio = 1.623, *p* < 0.001) and VD (odds ratio = 1.460, *p* = 0.010) by one degree significantly related to falls risk (see results in Table 4).

**Table 3:** Comparison of static SVV, dynamic SVV, and VD with balance assessment results.

Balance assessment tools (groups)	Static SVV		Dynamic SVV		VD	
	mean (95% Confidence Interval)	*p*	mean (95% Confidence Interval)	*p*	mean (95% Confidence Interval)	*p*
**TUG**		0.246		0.240		0.382
(1) low risk of falling	1.63 (0.01; 4.24)		5.50 (0.32; 12.33)		3.88 (2.67; 10.43
(2) medium risk of falling	3.39 (2.16; 4.62)		4.79 (3.47; 6.11)		1.41 (0.89; 1.93)
(3) high risk of falling	3.41 (2.46; 4.35)		7.53 (5.29; 9.77)		4.23 (2.03; 6.42)
**Tandem stance**		0.560		0.080		0.602
(1) low risk of falling	2.91 (1.93; 3.88)		4.93 (3.72; 6.14)		2.13 (1.29; 2.98)
(2) high risk of falling	3.44 (2.44; 4.45)		7.82 (5.36; 10.27)		4.41 (1.94; 6.89)
**Tinetti**		0.083		0.037		0.203
(1) moderate or low risk of falling	2.42 (1.57; 3.26		4.57 (2.97; 6.15)		2.16 (0.64; 3.68)
(2) high risk of falling	3.64 (2.70; 4.58)		7.66 (5.55; 9.76)		4.11 (2.01; 6.21)

*Abbreviations: TUG, Timed Up and Go; SVV, Subjective Visual Vertical; VD, Visual Dependence*.

**Table 4:** The relationship between the risk of falling according to the Tinetti test, the faller group, and the results of static SVV, dynamic SVV, and VD evaluations.

Predictor	Risk of falling according to the Tinetti test			Risk of falling according to the clinical history		
	Odds Ratio	*p*-value	95% Confidence Interval	Odds Ratio	*p*-value	95% Confidence Interval
Static SVV	1.426	0.090	0.946; 2.149	1.365	0.023	1.045; 1.784
Dynamic SVV	1.356	0.049	1.001; 1.835	1.623	<0.001	1.247; 2.114
VD	1.166	0.213	0.916; 1.486	1.460	0.010	1.093; 1.950

*Abbreviations: SVV, Subjective Visual Vertical; VD, Visual Dependence*.

### Comparison of the SVV Tests and Other Balance Assessment Tools in the Faller Group

We evaluated differences in tilts of static and dynamic SVV, VD values, and the results of other balance assessment tools. Older adults who had a high risk of falling, according to the Tinetti test results, had significantly higher tilts of dynamic SVV than those who had a low or medium risk (see results in Table 3). We subsequently evaluated the relationship between falls history and the results of static SVV, dynamic SVV tilts, and VD assessment, and compared this to the relationship between fall history and the Tinetti test outcomes. Univariate logistic regression analysis was applied for each quantitative predictor. We found that the increase in dynamic SVV tilt by one degree in the faller group was significantly related to falls risk (odds ratio = 1.356, *p* = 0.049) with a 1.4-fold increased odds of falling (see results in Table 4).

According to the 5TSTS test results, only two adults had a low risk of falling, and all others had a high risk of falling. Due to unequal groups, we did not compare the SVV test results between the grouped 5TSTS test results. There was no statistically significant difference in SVV error, for all conditions, across TUG and tandem stance tests (see results in Table 3).

### The Impact of Dizziness, Depression, and Mental State on SVV Tests and VD Results in the Faller Group

We correlated static and dynamic SVV tilts and VD values with dizziness (DHI), depression (GDS), and mental state evaluation (MMSE) scores but found no statistically significant relationship between these variables.

### Results of the SUS Assessment of the VIRVEST System in the Faller and Non-faller Groups

SUS scores indicated the degree to which the VIRVEST design was appropriate and easy to use from a participant’s point of view. SUS scores for the VIRVEST system were high, corresponding to a perceived rating of “excellent” in both groups (Table 5). There was no statistically significant difference in mean SUS scores between the two groups (*p* = 0.58).

**Table 5:** Results of the SUS assessment of the VIRVEST system in the faller and non-faller groups.

Measure	Mean ± SD	Median	Minimum	Maximum
SUS score in the	89.0 ± 10.7	90	60	100
non-faller group
SUS score in the	85.5 ± 15.7	90	35	100
faller group

*Abbreviations: SUS, System Usability Scale*.

## Discussion

We identified larger biases in static SVV, dynamic SVV, and VD values in older adults who experienced falls compared to age- and gender-matched controls. This finding hints at a deficit of otolithic function and visual-vestibular integration as a contributor to these falls.

Studies of SVV in older age subjects are inconsistent. Some studies show that SVV perception does not change with age (Baccini et al., 2014; Ashish et al., 2016; Zakaria et al., 2019), whilst others that SVV tilts increase with age (Jovanović, 2008). However, whilst one study found that static SVV perception was stable with age, dynamic SVV perception gradually increased with age (Kobayashi et al., 2002). In our study, the mean static SVV was 2.2 (*SD* = 1.7) degrees in the control group and was within the normal range for healthy people, despite the more advanced age in our group. Our findings support that the static SVV remains stable with age but that the dynamic SVV might be more affected [mean dynamic SVV of 3.3 (*SD* = 1.8)]. In the faller group, the mean static SVV was above the normal range, perhaps reflecting otolithic dysfunction. Dynamic SVV values in the faller group were higher than those in the control group, with a mean of 6.6 (*SD* = 4.5) degrees. The perception of spatial orientation depends on the brain’s integration of visual, vestibular, proprioceptive, and somatosensory signals. When one of these signals is disrupted, such as the vestibular signal, an individual starts compensating by relying more on the remaining cues (Medendorp et al., 2018). Based on our findings in the faller group, we propose a sensory reweighting in which the brain favors more reliable sensory cues (such as vision) to determine verticality perception. Such sensory re-weighting towards the visual system may be multifactorial, given the deterioration of sensory function across all modalities with age (Iwasaki and Yamasoba, 2015). However, the mean VD in the faller group was 3.4 (*SD* = 4.3) degrees, being less marked than in the dynamic SVV, perhaps due to a greater static SVV error in the faller group—a factor used to compute VD. We cannot differentiate whether abnormal dynamic SVV is a consequence of increased perceived fall risk, or causally related to fall risk, given the known influences of anxiety upon visual dependency, at least in young individuals (Bednarczuk et al., 2020).

Alberts et al. showed that with age, vestibular information is down-weighted, whereas visual weight is increased and that this shift in sensory reweighting is primarily due to an age-related increase in the noise of vestibular signals (Alberts et al., 2019). The results of our study demonstrate an additional interface given that the increased visual weight can also be associated with the risk of falls. Higher dynamic SVV tilts were statistically significantly related to a high risk of falling according to the Tinetti test results. SVV errors were however decorrelated from results on the TUG and tandem stance tests. The Tinetti test is widely used in older adults to assess mobility, balance, and gait, as well as to predict falls (Köpke and Meyer, 2006). The advantages of the Tinetti balance assessment tool are that it evaluates both balance and gait and has good interrater reliability and excellent sensitivity (Maki et al., 1994). Moreover, we found that the increase in the dynamic SVV tilt by one degree in the faller group was statistically significantly related with a 1.4-fold increased odds of falling when the risk of falls was evaluated with the Tinetti test. We further analyzed how the risk of falls was related to the results of static SVV, dynamic SVV tilts, and VD assessment compared to the history of falls. Across control and faller groups, we found that an increase in static SVV (odds ratio = 1.365, *p* = 0.023), dynamic SVV (odds ratio = 1.623, *p* < 0.001), and VD (odds ratio = 1.460, *p* = 0.010) tilt by one degree significantly related to falls risk.

Lord et al. investigated the relationship between VD and falls in their study. They measured SVV during the roll vection test with a dynamic background. The mean error in perception of the true vertical in the roll vection test was 6.6 degrees for the faller group and 3.6 degrees for the non-faller group (Lord and Webster, 1990). Barr et al. also evaluated a link between VD and falls using the roll vection test. Participants who reported multiple falls in the previous year had a significantly larger deviation from vertical in the roll vection test than those who reported no or only one fall (Barr et al., 2016). In previous studies, VD was measured irrespective of static SVV. Therefore, the concept of their VD matches our measurement of dynamic SVV, and the results are in line with ours. Using static and dynamic SVV measurements may be of practical utility to help guide the type of rehabilitation required for a given individual, whereby adults with the greatest errors on dynamic SVV tests (compared to static SVV) may benefit from additional visual context training, rather than only somatosensory training (Medendorp et al., 2018; Alberts et al., 2019).

The fact that no associations of SVV tilt and VD were observed with dizziness, depression, or cognitive function suggests that dynamic SVV may be an independent fall risk factor. Moreover, lower MMSE scores did not result in greater SVV errors, implying it is a valid test in this faller group cohort. Five fallers were unable to perform SVV tests using the VIRVEST system because they did not understand how to perform the test and were not included in the study so perhaps the exclusion criteria with a cut-off of 10 points for the MMSE was too low. On the other hand, some fallers had just 12 points on the MMSE score and were able to understand and perform the tests.

In the present study, using the novel mobile virtual reality system VIRVEST for subjective visual vertical and visual dependence assessment was employed for the first time in older people with and without falls. Excellent system usability scores of the VIRVEST system, both in fallers and non-fallers, demonstrated that VR-based SVV testing is suitable and comprehensible, even in older people. Currently, there is huge potential for new diagnostic and prognostic methods to detect deficits in multisensory integration (Lewis, 2015). Such methods should help track the quality of sensory systems across the life span or disease, apply risk factors, and indicate when older people may need additional care or training to maintain an active life (Medendorp et al., 2018). Falls in older individuals are however most often multifactorial in nature, including muscular strength, visual disturbances, neurological disturbance, proprioceptive loss, postural anxiety, and nutrition. We argue that visual dependency may be a further factor in fall risk that can be identified at the bedside, and has a potential intervention with physical rehabilitation strategies (Pavlou et al., 2004).

One limitation of our study is that we used retrospective fall data, which does not allow us to make conclusions about the cause of falls. Another limitation was the small sample size of the groups. However, the sample size far exceeds that typically used in studies exploring SVV in patients with neurological disorders (Dakin et al., 2018; Klatt et al., 2019; José Luvizutto et al., 2020). Given the subjective nature of the test and thus being a cognitive task, SVV may be more challenging to measure in patients with moderate or severe cognitive impairment (five individuals were unable to complete the test in our cohort due to poor task comprehension). Whilst this may represent a general issue with SVV, the VIRVEST system has not been specifically validated in patients with cognitive impairment. We were unable to formally exclude subtle impairments in hearing or vestibular function in patients as we were unable to carry out detailed audiovestibular function tests in the study setting.

In summary, this study, we evaluated the application of the virtual reality-based VIRVEST system SVV tests in older adults who had experienced falls. These adults manifested greater static and dynamic SVV tilts and VD values than the controls and had a greater risk of falls. The dynamic SVV test was the most useful test with high tilts that were statistically significantly related (according to the Tinetti test OR = 1.356, *p* = 0.049; according to falls history OR = 1.623, *p* < 0.001) to increased risk of falling. Given the high system usability scores, the VIRVEST system may be applicable in clinical settings for SVV testing and predicting falls in older adults.

## Data Availability Statement

The raw data supporting the conclusions of this article will be made available by the authors, without undue reservation.

## Ethics Statement

The studies involving human participants were reviewed and approved by Kaunas Regional Biomedical Research Ethics Committee. The patients/participants provided their written informed consent to participate in this study.

## Author Contributions

MT: data curation and writing—original draft. VU: conceptualization, methodology, and supervision. VL and GD: methodology and supervision. RK: data curation. DK: supervision and writing—original draft. IU: conceptualization, methodology, and writing—original draft. All authors contributed to the article and approved the submitted version.

## Conflict of Interest

The authors declare that the research was conducted in the absence of any commercial or financial relationships that could be construed as a potential conflict of interest.
